# Towards integrated malaria molecular surveillance in Africa

**DOI:** 10.1016/j.pt.2024.09.005

**Published:** 2024-10-29

**Authors:** Nsa Dada, Victoria J. Simpson, Lucas N. Amenga-Etego, Eniyou Oriero, Olivo Miotto, Mili Estee Torok, Elijah O. Juma, Nana Aba Williams, Shavanthi Rajatileka, Cristina V. Ariani, Jaishree Raman, Deus S. Ishengoma

**Affiliations:** 1 School of Life Sciences, Arizona State University, Tempe, AZ, USA; 2 Center for Fundamental and Applied Microbiomics, The Biodesign Institute, Arizona State University, Tempe, AZ, USA; 3 Genomic Surveillance Unit, Wellcome Sanger Institute, Hinxton, Cambridgeshire, UK; 4 West African Centre for Cell Biology of infectious Pathogens, University of Ghana, Accra, Ghana; 5 The Medical Research Council Unit The Gambia at the London School of Hygiene and Tropical Medicine, Fajara, Gambia; 6 Centre for Tropical Medicine and Global Health, Nuffield Department of Medicine, University of Oxford, Oxford, United Kingdom; 7 Mahidol-Oxford Tropical Medicine Research Unit, Faculty of Tropical Medicine, Mahidol University, Bangkok, Thailand; 8 The Bill & Melinda Gates Foundation, London, United Kingdom; 9 The Pan-African Mosquito Control Association (PAMCA), KEMRI HQ, Nairobi, Kenya; 10 MESA, the malaria knowledge hub, Barcelona Institute for Global Health (ISGlobal), Hospital Clinic-Universitat de Barcelona, Barcelona, Spain; 11 Centre for Emerging Zoonotic and Parasitic Diseases, National Institute for Communicable Diseases, Johannesburg, South Africa; 12 Wits Research Institute for Malaria, Faculty of Health Sciences, University of Witwatersrand, Johannesburg, South Africa; 13 UP Institute for Sustainable Malaria Control, Faculty of Health Sciences, University of Pretoria, Pretoria, South Africa; 14 National Institute for Medical Research, Dar es Salaam, Tanzania.; 15 Harvard T.H Chan School of Public Health, Boston, MA, USA; 16 Kampala International University in Tanzania, Dar es Salaam, Tanzania

**Keywords:** malaria, molecular surveillance, genomics, genomic surveillance, regional hubs

## Abstract

Integrated malaria molecular surveillance (iMMS) systems are essential for Africa’s expanding malaria genomics initiatives. We highlight a few initiatives and demonstrate how iMMS can support evidence-based decisions and policies for National Malaria Programs and other malaria control stakeholders. We conclude with key considerations for advancing these malaria genomics initiatives towards sustainable iMMS.

## The need for integrated malaria molecular surveillance (iMMS)

**Figure 1.**
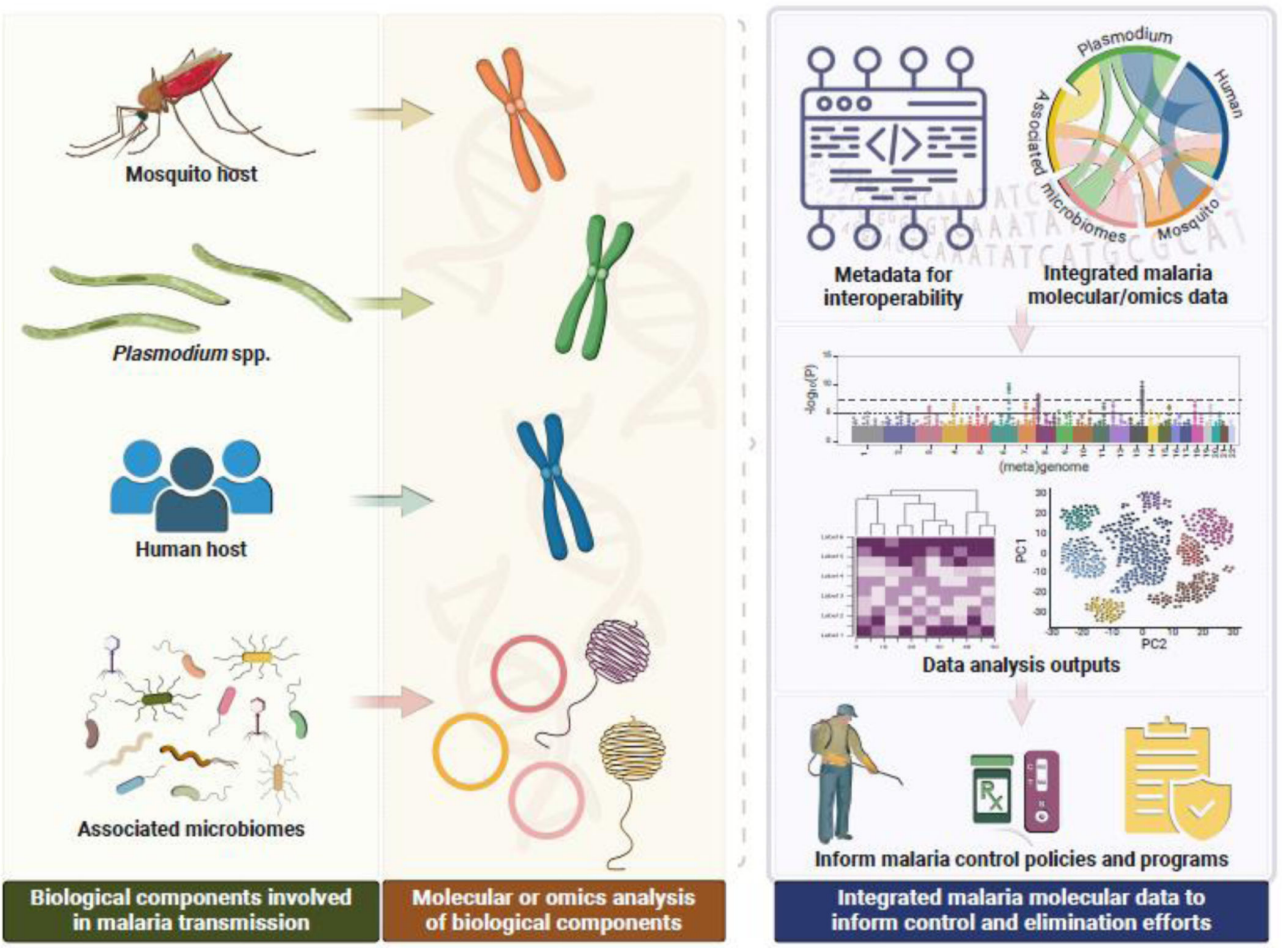
Overview of Integrated Malaria Molecular Surveillance (IMMS). An illustration of how the integration of molecular and omics studies of each biological component of malaria transmission (i.e., *Plasmodium* parasites, mosquito and human hosts) can provide comprehensive evidence to inform malaria surveillance, control programs, and policies.

Advancements in molecular biology have led to an expansion of genomic surveillance of diseases including malaria [1–4] as exemplified by the projects within the Malaria Genomic Epidemiology Network, a worldwide network of researchers collaborating on large-scale studies that integrate epidemiology with genomic analysis to understand how genetic variation in humans, *Plasmodium* parasites, and *Anopheles* mosquitoes influences malaria transmission and the ongoing evolutionary interactions among them. These studies include, among others, projects focused on the genetic diversity of *Plasmodium* species and their evolution of resistance to frontline drugs, as well as the population genetics of *Anopheles* mosquitoes ^i^ with use cases for National Malaria Programs ^ii^. The growing use of molecular tools in malaria research and surveillance is expanding the range of strategies for surveillance and control [2,4] and our understanding of the various biological components that interact to sustain malaria transmission—human host, malaria parasites, mosquito vectors, and the microbes that are associated with each of these components [5,6]. As seen with the COVID-19 pandemic [7,8], interoperable data from multiple sources using robust integrated molecular surveillance are essential for the sustainable control and elimination of diseases. The establishment of integrated malaria molecular surveillance (iMMS) (Figure 1) at national levels will enable country-led decision-making informed by the molecular precision of how interacting biological components respond to specific malaria control interventions at a granular level. A critical prerequisite for success is the establishment of systems that are sustainable and employ interoperability standards that allow the data to serve each country while also capturing the diversity of interactions that sustain malaria transmission across regions. Efforts are urgently needed to establish the requisite human and infrastructural capacity to deploy, utilize, and sustain iMMS platforms.

## Establishing and sustaining iMMS by leveraging regional malaria research hubs

**Figure 2.**
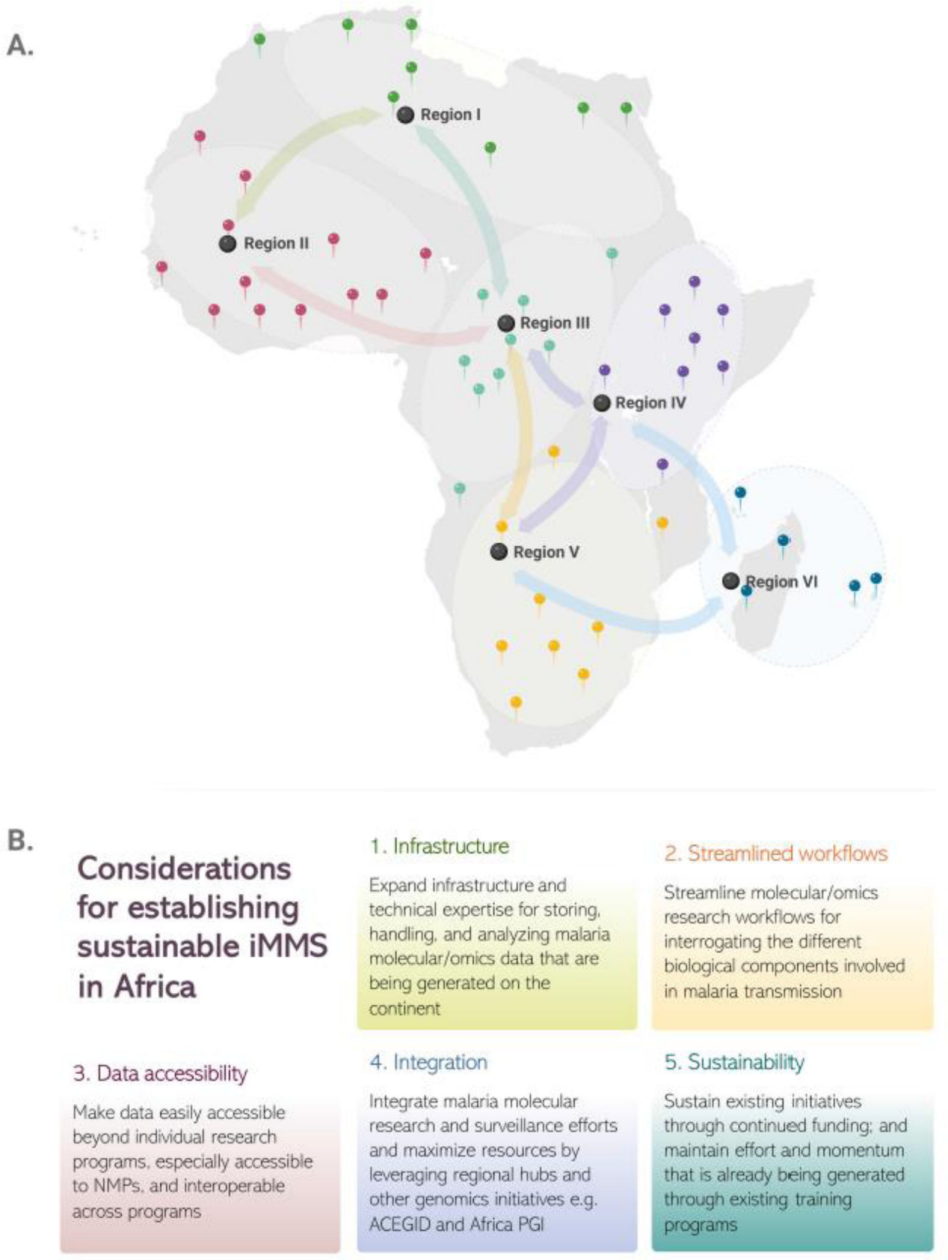
Considerations for establishing sustainable Integrated Malaria Molecular Surveillance (iMMS) in Africa. (A) An illustration of how regional hubs can be leveraged to maximize malaria molecular and genomics efforts on the continent. The regional hubs would ideally include individual laboratories or initiatives in various parts of the continent that have a regional catchment to facilitate cross-country collaboration and resource sharing. This coordinated efforts across different countries will ensure maximum iMMS coverage and impact on malaria control. For illustrative purposes, the map indicates the interacting network (arrows) of hypothetical locations of regional hubs in Africa (black location markers), each coordinating activities across the countries that it serves (colored bubbles with correspondingly colored geo pins). (B) Key considerations for establishing sustainable iMMS in Africa. NMPs stands for national Malaria Programs; ACEGID—African Center of Excellence for Genomics of Infectious Diseases; and Africa PGI—Africa Pathogen Genomics Initiative, flagship initiative of the Africa Centers for Disease Control and Prevention. Panels A & B of this figure were created using BioRender with some elements obtained from the Noun Project ^xvii^ and the Integration and Application Network ^xviii^.

One of the strategies that is being considered to strengthen capacity and support for iMMS implementation is the establishment of regional hubs (Figure 2A). These hubs would include regional research or public health reference laboratories with strong capacity to support surveillance at multinational and regional scales. The hubs would facilitate the integration of efforts and ensure that data from various countries can be utilized within regional, continental, and even global contexts. Through active engagement with policy makers and National Malaria Programs, the hubs would work towards generating pertinent data that are used to inform policy and decision making (Figure 1). To ensure sustainability, each hub would also catalyze their own discrete but complementary regional networks and facilitate knowledge exchange within the network [9–11].

Programmatic application of genomics research is growing in Africa [12], but iMMS platforms supporting the generation, processing, storage, analysis, and integration of malaria molecular data, particularly genomics data, as well as regional centers of genomic surveillance are limited and not widely implemented. Here, we highlight recent malaria genomics initiatives in Africa with potential for iMMS and an example of a successful initiative from the Greater Mekong Subregion that we could learn from. We also discuss existing support for malaria genomics research on the continent and provide considerations for establishing integrated malaria genomics efforts toward sustainable iMMS in Africa. Our goal is to facilitate discussions and actions towards integrating malaria genomics efforts on the continent and demonstrate how iMMS can support evidence-based decisions and policymaking by National Malaria Programs and other stakeholders. The initiatives and support for malaria molecular research in Africa highlighted here are based on the outcomes of a symposium we convened at the 72nd annual meeting of the American Society of Tropical Medicine and Hygiene held in October 2023, in Chicago, USA ^iii^, to address this topic. See supplemental information for full report of the symposium.

## Examples of malaria genomics initiatives in Africa with potential for iMMS

### Integrated malaria parasite and vector molecular surveillance in Ghana

The Ghana National Malaria Elimination Program has implemented several high-impact interventions, including indoor residual spraying, insecticide-treated nets, and seasonal malaria chemoprevention, leading to varying levels of success in reducing malaria transmission [13] However, significant transmission heterogeneity persists, driven by shifts in intervention effectiveness, highlighting the need for innovative tools. The Integrated Malaria Parasite and Vector Molecular Surveillance project ^iv^, supported by a grant from the Bill & Melinda Gates Foundation, has facilitated comprehensive molecular surveillance across Ghana’s 16 administrative regions, focusing on mosquito and parasite genomics. Early results indicate widespread pyrethroid resistance in *Anopheles gambiae*, with new drug resistance alleles emerging in malaria parasites but no validated artemisinin-resistant mutations detected to date. Ongoing efforts include phenotyping and sequencing 500 malaria vectors per region and optimizing in-country genomic assays to effectively monitor and respond to emerging drug and insecticide resistance trends.

### Establishing a regional hub for malaria genomic surveillance in The Gambia

A proof-of-concept study aimed at using genomics and bioinformatics tools to improve malaria surveillance was piloted in The Gambia, and in collaboration with partner countries in the West-African sub-region, has further expanded to establishing a regional genomic surveillance hub at the Medical Research Council Unit, the Gambia ^v^. Health facility-based surveys were conducted during the peak malaria transmission seasons from 2019 to 2022, and the Malaria Genomic Epidemiology Network’s Amplicon toolkit ^vi^ for the genomic surveillance of malaria parasites was established for in-house sample processing. Results from the Gambia, Guinea, and Sierra Leone revealed a low frequency of *kelch*13 mutations associated with artemisinin partial resistance but high frequencies of chloroquine and pyrimethamine resistance mutations. These findings underscore the importance of expanding malaria genomic surveillance beyond the national level to track and respond to regional resistance patterns. The teams are scaling up operations as a regional hub, to increase data generation.

### A Pan-African bioinformatics initiative for malaria vector research and surveillance

‘Global North-South’ collaborations have expanded sequencing capacity in Africa, but limited computing infrastructure and technical expertise have hindered full utilization of the resulting genomic data. This gap compromises the impact of genomics on malaria vector surveillance and control. To address this, a Pan-African bioinformatics initiative was launched, coordinated by the Pan-African Mosquito Control Association ^vii^, to build sustainable bioinformatics capacity for malaria vector genomics research. The initiative’s goals are to democratize bioinformatics resources, develop critical technical expertise, ensure data sovereignty on the continent, and work with National Malaria Programs to integrate genomics research outputs into malaria control and elimination strategies. Achievements since 2022 include a Training-of-Trainers Bioinformatics Fellowship program which has trained four bioinformaticians, and an ‘analysis-ready’ data training program, with open access training resources ^viii^, reaching over 260 scientists across 40 African countries. Additionally, high-performance computing infrastructure has been established to support data storage, processing, and utilization on the continent. Sustaining these efforts will require prompt adoption of rapidly evolving technologies and strengthening collaborations between National Malaria Programs and other relevant stakeholders across the continent.

## Accessible informatics tools for enhancing public health value of malaria molecular surveillance data: Lessons from the Greater Mekong Subregion

Translating malaria genomics data into actionable evidence requires specialized expertise outside the remit of National Malaria Programs, and genomic surveillance must be contextualized to effectively monitor intervention impact. To address the challenges of translating genomic data and support *Plasmodium falciparum* elimination, the GenRe-Mekong project [14] partnered with National Malaria Programs in the Greater Mekong Subregion to develop informatics tools ^ix^. These tools enhance malaria genomic surveillance by predicting drug resistance, integrating genomic data with geographical metadata, and generating statistics while managing data complexity. Additionally, intuitive visualizations with “traffic light” schemes simplify data interpretation. An example of such tools is the *grcMalaria* software [15] ^x^ that is accessible with minimal programming experience, with web and mobile applications now being developed to further enhance accessibility and support cross-border surveillance efforts.

## Support for malaria genomics research in Africa and future outlook for sustaining iMMS on the continent

Sustainable funding, human and infrastructure capital for malaria genomics research, along with resource development and sharing are crucial for implementing and sustaining iMMS in Africa. Here we highlight some of the ongoing support for malaria genomics research that can be leveraged for establishing and sustaining iMMS in Africa:

- The Bill & Melinda Gates Foundation has committed funding to initiatives that build human and infrastructure capital for malaria genomics research in Africa. These initiatives, some of which are exemplified above, have great potential for establishing iMMS on the continent. The committed funding and initiatives aim to catalyze further action. As such, researchers, National Malaria Programs, and other stakeholders—like the African Pathogen Genomics Initiative ^xi^, African Center of Excellence for Genomics of Infectious Diseases ^xii^, or Pathogens Diversity Network Africa ^xiii^—must exercise their agency in identifying sustainable means for maintaining these investments and establishing iMMS on the continent. This may involve lobbying for local, national, regional, or even continent-wide funding mechanisms, as well as forming strategic partnerships among research institutions, National Malaria Programs, policymakers, and government entities to leverage these funding opportunities. For example, the African Pathogen Genomics Initiative ^xi^ through the African Union, could lead efforts in lobbying African governments to devote a percentage of the GPD towards genomics surveillance, particularly in the context of endemic pathogens like the malaria parasite and its vectors.
- The Pan-African Mosquito Control Association has created a network ^xiv^ for malaria vector genomics surveillance on the continent that is aimed at generating actionable data for decision-making by National Malaria Programs. This network brings together various African institutions working on vector control, with medical entomology and genetics expertise to support continent-wide sampling and genetic analysis of prevalent malaria vector species. This is in addition to partnering with global experts to expand bioinformatics capacity on the continent. These efforts are expanding technical expertise to support the analysis of data generated from malaria vector genomics projects across Africa.
- The Wellcome Sanger Institute’s Genomic Surveillance Unit ^xv^ in collaboration with partners in Africa, such as the Pan-African Mosquito Control Association, provides in-country assistance for generating malaria parasite and vector genomics data. Through a decentralized approach, the in-country support is provided for the end-to-end process from sample collection to data generation including standardized protocols for sample and metadata collection, standardized laboratory protocols, support with laboratory set-up and establishing laboratory workflows, procurement support where feasible, tailored training for individual laboratories, bespoke laboratory train-the-trainer programs, ongoing troubleshooting and technical support, as well as sample and data analysis. The approach taken is partner-led, and teams at the Genomic Surveillance Unit work together with individual partners to develop bespoke implementation and support plans and in close collaboration with laboratory teams tailor protocols to an individual laboratory’s requirements and available resources. This ensures the training provided, the support given, and any resources developed is fit-for-purpose in each setting. The Genomic Surveillance Unit has supported several laboratories in Africa and Southeast Asia and is committed to continuing its collaborative efforts to establish genomic surveillance capacity in endemic countries.
- MESA—the Malaria Knowledge Hub ^xvi^ assesses the landscape of malaria research and evidence relevant to malaria control and elimination to inform decisions. This Knowledge Hub offers a platform to identify and bring the key players and institutions involved in policy-relevant malaria genetic epidemiology studies to address the unmet challenges in bridging the gap between the use of molecular and genomics surveillance approaches to fight malaria, promote the sharing of knowledge, resources, and best practices. To bridge knowledge gaps in malaria molecular and genomic research as well as gaps between malaria genomic research and control policy, the Malaria Knowledge Hub is compiling emerging research evidence and ensuring that ensuring that these pieces of evidence promptly informs decision-making to maximize malaria control impact.

While progress is being made, a significant amount of work remains to ensure full coverage of malaria molecular and genomics efforts and its benefits, including iMMS, across the African continent. Overall, considerations that stakeholders agree on for establishing and sustaining iMMS in Africa include expanding malaria genomics infrastructure and expertise, streamlining workflows, improving data accessibility and interoperability, integrating efforts across the continent, including leveraging other genomics surveillance initiatives on the continent like the African Pathogen Genomics Initiative ^xi^, African Center of Excellence for Genomics of Infectious Diseases ^xii^, or Pathogens Diversity Network Africa ^xiii^, as well as sustained funding (Figure 2B).

Looking ahead, some practical ways to implement these considerations include collaborating to develop, at a minimum: (i) well-structured guidelines for each stage of the malaria genomics research workflow including how to navigate supply chain issues. This should be accompanied by training that could be agnostic of research questions to ensure wide adaptation and use (ii) user friendly “plug-and-play” data analysis tools that require minimal technical expertise to use (iii) a strategy to ensure that these resources and tools remain relevant and do not become obsolete. This will require sustainable funding, perhaps through buy-in from local governments and institutions.

## Supplementary Material

1

## Footnotes

i Projects under the Malaria Genomic Epidemiology Network (MalariaGEN): https://www.malariagen.net/about/projects/#

ii Use cases for National Malaria Control Programs: https://www.malariagen.net/resources/use-cases-for-nmcps/

iii 72^nd^ ASTMH Meeting Program Book. Symposium 88, Integrated Malaria Molecular Surveillance (iMMS) in Africa: Current Initiatives and Future Direction : https://www.astmh.org/getmedia/1be53405–65c3–4856-8b70–7ea59b4cc8fc/ASTMH-2023-Annual-Meeting-Program-Book-All-Pages.pdf

iv The Integrated Malaria Parasite and Vector Molecular Surveillance project: https://www.malariagen.net/partner_study/1339-vo-gh-amenga-etego/

v Establishing Regional Hubs for Genomic Surveillance in West Africa: https://fundingawards.nihr.ac.uk/award/NIHR134717

vi Amplicon toolkit: https://www.malariagen.net/resources/amplicon-sequencing-toolkit/

vii PAMCA-MalariaGEN bioinformatics initiative: https://www.pamca.org/en/projects/view/4/anopheles-genomics-project-phase-ii-building-sustainable-capacity-in-vector--genomics-and-bioinformatics-in-africa

viii PAMCA-MalariaGEN bioinformatics training resources: https://anopheles-genomic-surveillance.github.io/home.html

ix GenRe-Mekong project: https://genremekong.org/tools

x grcMalaria R package: https://genremekong.org/tools/grcmalaria-r-package-user-guide

xi Africa pathogen Genomics Initiative: https://africacdc.org/africa-pathogen-genomics-initiative-africa-pgi/

xii African Center of Excellence for Genomics of Infectious Diseases ACEGID: https://acegid.org/

xiii Pathogens Diversity Network Africa: https://pathogens-dna.org/en/home-4

xiv PAMCA Anopheles 1000G genomic project: https://www.pamca.org/en/projects/view/3/anopheles-1000g-genomic-project:-opportunity-to-fill-data-gaps

xv Genomic Surveillance Unit, Wellcome Sanger Institute: https://www.sanger.ac.uk/collaboration/genomic-surveillance-unit/

xvi MESA—the malaria knowledge hub: https://mesamalaria.org/

xvii Noun Project: www.thenounproject.com

**xviii:** Integration and Application Network: www.ian.umces.edu/media-library
