# Supplementary material for: Towards integrated malaria molecular surveillance in Africa
Source: Trends Parasitol. Author manuscript; Available in PMC 2025 Apr 10. (PMC11984316; doi:10.1016/j.pt.2024.09.005)
Supplement: 1 [file NIHMS2027187-supplement-1.docx]

# **Supplemental information**

# Towards integrated malaria molecular surveillance in Africa

### Nsa Dada^1,2^*, Victoria J. Simpson^3^, Lucas N. Amenga-Etego^4^, Eniyou Oriero^5^, Olivo Miotto^6,7^, Mili Estee Torok^8^, Elijah O. Juma^9^, Nana Aba Williams^10^, Shavanthi Rajatileka^3^, Cristina V. Ariani^3^, Jaishree Raman^11,12,13^, Deus S. Ishengoma^14,15,16^*

*^1^School of Life Sciences, Arizona State University, Tempe, AZ, USA*

*^2^Center for Fundamental and Applied Microbiomics, The Biodesign Institute, Arizona State University, Tempe, AZ, USA*

*^3^Genomic Surveillance Unit, Wellcome Sanger Institute, Hinxton, Cambridgeshire, UK*

*^4^West African Centre for Cell Biology of infectious Pathogens, University of Ghana, Accra, Ghana*

*^5^The Medical Research Council Unit The Gambia at the London School of Hygiene and Tropical Medicine, Fajara, Gambia*

*^6^Centre for Tropical Medicine and Global Health, Nuffield Department of Medicine, University of Oxford, Oxford, United Kingdom*

*^7^Mahidol-Oxford Tropical Medicine Research Unit, Faculty of Tropical Medicine, Mahidol University, Bangkok, Thailand*

*^8^The Bill & Melinda Gates Foundation, London, United Kingdom*

*^9^The Pan-African Mosquito Control Association (PAMCA), KEMRI HQ, Nairobi, Kenya*

*^10^MESA, the malaria knowledge hub, Barcelona Institute for Global Health (ISGlobal), Hospital Clinic-Universitat de Barcelona, Barcelona, Spain*

*^11^Centre for Emerging Zoonotic and Parasitic Diseases, National Institute of Communicable Diseases, Johannesburg, South Africa*

*^12^Wits Research Institute for Malaria, Faculty of Health Sciences, University of Witwatersrand, Johannesburg, South Africa*

*^13^UP Institute for Sustainable Malaria Control, Faculty of Health Sciences, University of Pretoria, Pretoria, South Africa*

*^14^National Institute for Medical Research, Dar es Salaam, Tanzania.*

*^15^Harvard T.H Chan School of Public Health, Boston, MA, USA*

*^16^Kampala International University in Tanzania, Dar es Salaam, Tanzania*

***Correspondence**: [Nsa.Dada@asu.edu](mailto:Nsa.Dada@asu.edu) (Nsa Dada); [deusdedith.ishengoma@nimr.or.tz](mailto:deusdedith.ishengoma@nimr.or.tz) (Deus Ishengoma)

# Background

At the 72nd Annual meeting of the American Society of Tropical Medicine and Hygiene (ASTMH) held in Chicago, USA, we convened a symposium bringing together a team of genomics experts, bioinformaticians, data analysts, funders, and policy makers to discuss and address cross-cutting questions to deepen our understanding of the impacts of different malaria interventions and craft a path towards integrated genomics efforts with a focus on iMMS. By addressing these challenges collectively, our goal was to facilitate, among many other factors, the development of key operational questions and offer recommendations for the implementation of evidence-based decisions and policymaking by NMCPs and other stakeholders that is based on iMMS.

The symposium brought together experts actively working in different areas of iMMS. The presentations showcased ongoing efforts to develop sustainable workflows and initiatives for generating malaria genomic data and expanding capacity for data handling and analysis, as well as collective efforts towards effectively implementing iMMS and creating regional hubs across Africa. The presentations demonstrated how a harmonized implementation of genomic surveillance of malaria can address challenges including material procurement, implementing pertinent training, as well as data interoperability. There were specific examples of iMMS implementation in Ghana, The Gambia, Mali, and Vietnam, that included how to integrate with NMCPs to demonstrate relevant use cases, and how a regional network for genomic surveillance can be built.

In addition to the presentations, a diverse group of panelists composed of experts and representatives from various agencies including the Bill & Melinda Gates Foundation, the Pan-African Mosquito Control Association, the Malaria Eradication Scientific Alliance, and the Wellcome Sanger Institute, discussed and highlighted pressing issues and perspectives on effective implementation of iMMS in malaria endemic countries. Below, we provide an overview of each component of the symposium and summarize take home messages and future directions for iMMS in Africa.

#

# Overview of presentations

***Establishment and implementation of an Integrated Malaria Parasite and Vector molecular Surveillance in Ghana (IMPAVES-Ghana) by Lucas Amenga-Etego from the West African Centre for Cell Biology of infectious Pathogens (WACCBIP), University of Ghana, Accra, Ghana***

Over the years, the deployment of strategic high impact interventions by the Ghana National Malaria Elimination Program (NMEP) such as indoor residual spraying, long lasting dual insecticide treated nets, larval source management, seasonal malaria chemoprevention (SMC), perennial malaria chemoprevention (PMC), and intermittent preventive treatment (iPTp), has resulted in marginal to significant reductions in malaria transmission. However, the impact is a massive transmission heterogeneity marked by unstable parasite prevalence that is driven by shifts in transmission as a result of variable response to these interventions. These indicate that more innovative tools and interventions are urgently needed to eliminate malaria in Ghana.

Integrated Malaria Parasite and Vector Molecular Surveillance (IMPAVES) is a project that has been established in Ghana with the support of the Bill & Melinda Gates Foundation to build and expand the capacity for and implement iMMS. IMPAVES aims to establish integrated malaria parasite and vector surveillance using genomics and bioinformatics tools across the 16 geopolitical regions of Ghana. The project is currently conducting deep mosquito and parasite sampling in 36 community facilities across these regions to augment existing pilot data which shows that pyrethroid resistance mediated by the L995F (“kdr west”) mutation[S1] is fixed in *Anopheles gambiae* across Ghana but not in *An. coluzzii* populations. The pilot data shows a combination of new alleles (V402L+I1527T) rising in southern populations.

Through IMPAVES, the team will phenotype and sequence 500 malaria vectors per transmission zone to generate genomic data that will help explain the role of these gnome-wide signals in mediating resistance to specific insecticides. The team is also using the MalariaGEN Amplicon toolkit[S2] to genotype known antimalarial drug resistance mutations and generate whole genome data for population genetic analysis. Additionally, further optimization of the Oxford Nanopore Technology (ONT)-based amplicon assays for in-country determination of drug resistance mutations as well as whole genome sequencing of malaria parasites is ongoing. Results so far show no validated kelch13[S3] artemisinin resistant mutations. However, *Pfdhp*s 540E, 581G and 613S mutations associated with resistance to sulfadoxine, a component of sulfadoxine-pyrimethamine, have been found at low frequencies (<5%). These mutations need to be closely tracked because a higher frequency has the potential of reducing the effectiveness of sulfadoxine-pyrimethamine which is currently used in NMEP’s strategic high impact interventions.

***Establishing a regional hub for malaria genomic surveillance in The Gambia by Eniyou Cheryll Oriero from the Medical Research Council Unit The Gambia at LSHTM, Fajara, Gambia***

A progressive rise in resistance to the drugs and insecticides used for malaria control presents a major threat to sustainable malaria control across the African region. Drug and insecticide resistance increases the economic costs and reduces the effectiveness of malaria control in Africa and could reverse the gains that have been made in malaria control over the past 20 years. National malaria control programs (NMCPs) can benefit from genomic surveillance to monitor resistance, implement and assess the impact of control measures, and provide early warning if interventions are causing resistance to rise to unacceptable levels. Since knowledge of genetic markers and genetic epidemiology are outside the domain of expertise of most NMCPs, genomic surveillance hubs with subject matter expertise can support NMCP-managed activities in different countries and integrate data generated into regional analyses.

A proof-of-concept study aimed at using genomic and bioinformatics tools to improve malaria surveillance was piloted in The Gambia and was further expanded to partner countries in the West-African sub-region. Working closely with government stakeholders, Research Scientists at the Medical Research Council Unit The Gambia (MRCG) carried out health facility-based surveys during the peak malaria transmission seasons from 2019 to 2022. End-to-end implementation of genomic surveillance for malaria parasites using the MalariaGEN Amplicon toolkit[S2] was established at MRCG for sample processing in-house.

The results so far show that while there were low-frequency single nucleotide polymorphisms (SNPs) in Kelch 13 gene[S3], none of the SNPs that are associated with artemisinin partial resistance (ART-R) were observed in The Gambia. On the other hand, the chloroquine resistance haplotype CVIET[S4] was observed at a high frequency (>60%), and the triple *dhfr* mutants (51I, 59R and 108N)[S5] for resistance to pyrimethamine was almost fixed at frequencies ≥95% in the population. High frequencies of mutant alleles were also observed in one locus of the *dhps* gene (A437G)[6] associated with sulfadoxine resistance. The K540E[S6] mutant allele, also associated with sulfadoxine resistance, was also observed, but at low frequency in the eastern part of The Gambia. Similar patterns of drug resistance were observed in two partner countries (Guinea and Sierra Leone) apart from no K540E observed in Guinea and both K540E and K540N observed in Sierra Leone.

These results demonstrate the need for increased genomic surveillance across the sub-region to monitor parasite adaptations to current interventions.

***A Pan-African bioinformatics initiative for malaria vector research and surveillance by Nsa Dada from Arizona State University, Arizona, USA***

‘Global North-South’ collaborative initiatives have allowed for easier access to sequencing infrastructure, resulting in increasing sequencing capacity on the African continent[S7,S8]. However, the large amounts of data being generated are currently not being fully analyzed on the continent[S9–S11] or utilized to their full potential because the necessary computing infrastructure and technical expertise are limited. This represents a major gap in the utility and application of genomics. With increasing sequencing capacity on the continent, the use of genomic data for molecular surveillance of malaria parasites and vectors is also gaining popularity[S12–S14], but a critical mass of researchers who can adequately apply this approach to malaria vector surveillance and control is needed to reap and sustain its benefits.

A Pan-African bioinformatics initiative for malaria vector genomics research and surveillance was established to meet this critical need, with the goal of developing a strong foundation for sustainable bioinformatics capacity on the continent. Our objectives were to: (i) Democratize bioinformatics resources for vector genomics research on the continent (ii) Establish the minimum capacity required for processing and utilizing the data generated from vector genomics research on the continent via developing a critical mass of technical expertise and a sense of comradery to facilitate south-south collaborations and (iii) Facilitate agency in research while underscoring data sovereignty and ensuring that these efforts are sustained.

To ensure democratization of these resources, the Pan-African Mosquito Control Association (PAMCA)[S15] coordinates the initiative and provides oversight. PAMCA is a member-based professional body uniting stakeholders in vector control, disease elimination, and public health, with a core mandate in capacity building, collaboration, knowledge management, gender empowerment, and governance structures for the control and elimination of vector-borne diseases in Africa and globally.

To address the remaining objectives, we designed the initiative to leverage ongoing malaria vector genomics research on the continent facilitated by PAMCA in collaboration with MalariaGEN. As at the time of presenting these outcomes (October 2023), the initiative has so far established:

A Training-of-trainers (ToT) bioinformatics Fellowship program which encompasses a more specialized training for trainers who would subsequently develop and lead bioinformatics training programs for vector genomics research on the continent. So far, two cohorts of ToT Fellowships have been implemented with four Fellows trained and now providing bioinformatics support to various vector genomics research across the continent.

A training program on ‘analysis-ready’[S16] data to equip a critical mass of vector genomics researchers with pertinent skills for interrogating and analyzing vector genomics data. So far, we have completed two iterations of this training program wherein over 140 scientists from at least 40 African countries have been trained with resources openly available online for self-paced learning to extend the benefits beyond formally enrolled participants.

High performance computing infrastructure to cater to the computational needs arising from the extensive malaria genomics data being generated and emphasizing data sovereignty are urgently needed. We have acquired and installed high-performance computing (HPC) physical servers (Cloud HPC On Premise Edition), currently hosted by PSSC Labs, CA, USA. Specifications include 196,992 HPC cores, 120 TB storage, 10 GigE high-speed network communication, and a public network connectivity of at least 100 Mbps shared connection. These HPC servers are currently dedicated to malaria vector genomics data storage and processing, with future considerations for expansion beyond malaria vector genomics data and extension to cloud computing.

Engagements with NMCPs is critical to ensure that outputs of this initiative feed back into malaria control programs to inform policies and control efforts. At this stage, efforts are being placed on ensuring accessibility and integration of malaria vector genomics research data into NMCPs’ toolbox.

To sustain an initiative like this, we need to keep up with rapidly evolving omics and bioinformatics technologies; ensure active integration of research outcomes into the data portfolio of NMCPs; as well as work with other initiatives to integrate malaria genomics research efforts on the continent.

***Increasing Public Health Value of Malaria Surveillance Data by Olivo Miotto from the University of Oxford, Bangkok, Thailand***

Genetic surveillance provides public health authorities with actionable intelligence to inform decisions and help evaluate interventions. However, the analysis and translation of genetic data into knowledge meaningful to public health experts presents numerous challenges. First, data in the form of variants and alleles are difficult to interpret without training in genetics and parasite biology, and predicting clinical significance requires thorough familiarity with current scientific literature. Second, deriving summary statistics from genetic data presents technical challenges, such as aggregating samples geographically, or handling missing genotypes and multiclonal infections, and as such requires genetic epidemiology knowledge and programming skills. Finally, genetic surveillance needs to be functionally contextualized into public health activities- put simply, NMCPs need to monitor intervention impact, rather than allele frequencies.

The GenRe-Mekong project has partnered with NMCPs in the Greater Mekong Subregion since 2017, providing genetic surveillance to support *Plasmodium falciparum* elimination, and has addressed several of the aforementioned challenges. Informatics tools were developed to (i) augment genetic data with predictions of resistance levels to multiple drugs based on rules derived from literature searches; (ii) aggregate samples into units meaningful to NMCPs using structured geographical metadata; and (iii) derive statistics while accounting for missing data and complex infections.

To facilitate interpretation, intuitive visualization paradigms were introduced, such as “traffic light” color schemes to represent resistance prevalence. To support a broader range of needs, GenRe-Mekong conducted formal analyses of genetic epidemiology activities and their mapping to NMCP use cases[S17], resulting in several additional analyses. Genetic barcodes are used to estimate genetic diversity and site connectedness, which are informative of transmission intensity and gene flow, and to cluster samples by similarity. Mapping geographical clusters distribution was found particularly informative in outbreak analyses.

To make these analyses available to country partners, the publicly available *grcMalaria* software library[S18] was built to produce visualizations from data delivered by GenRe-Mekong, using R language scripts. Coding requirements are minimal, and the tutorials and documentation provided ensure rapid results without prior programming experience. To maximize accessibility, a Web application is under development, to produce maps and allow data download through simple point-and-click interfaces. *grcMalaria*[S19] accepts data in a well-documented format, which facilitates translation from other sources, such as the global MalariaGEN Pf7 dataset[S20], or data from surveillance projects in Africa. Merging multiple datasets allows for aggregated analyses–a critical feature for cross-border surveillance.

# Summary of the panel discussion session

## Highlights from panelists

***Need for sustainable funding for iMMS in Low- and Middle-Income Countries (Estee Torok from the Bill and Melinda Gates Foundation):*** The Bill & Melinda Gates Foundation[S21] has invested in building human capacity to sustain iMMS investments in Africa, and is committed to supporting low- and Middle-income countries (LMICs) via funding in establishing and sustaining iMMS. The funding for establishing iMMS on the continent is intended to catalyze further action. As such, NCMPs, their partners, and other stakeholders are encouraged to exercise their agency and identify sustainable means for maintaining investments in iMMS.

***Building and sustaining vector genomics capacity in Africa through PAMCA (Elijah Juma from the Pan African Mosquito Control Association, PAMCA):*** In realizing the need for vector genomics and bioinformatics skills on the continent, PAMCA, in collaboration with other organizations and leading global experts, has established an initiative to expand this capacity on the continent. This is being accomplished through training programs and a new bioinformatics fellowship program. As the bioinformatics fellows complete their fellowships, they are integrated into malaria vector genomics projects across the continent to provide technical expertise and support to African research institutions and NMPs.

***MESA’s role in supporting iMMS in Africa (Nana Williams Aba from MESA, the malaria knowledge hub):*** MESA, the malaria knowledge hub[S22], emerged from the overarching concept of the malaria eradication agenda (malERA)[S23] in 2011. Over the years, MESA has grown to become a powerful platform, convening the malaria community to collectively address and strategize solutions based on the latest evidence, research, and continuous learning. The use of iMMS approaches to derive epidemiologically actionable information for malaria control and elimination is vital for surveillance efforts by NMCPs, spanning individual countries and regions across malaria-endemic regions in Africa. MESA plans to support iMMS activities via distinct yet interconnected approaches:

- Assessment of the landscape of ongoing research on iMMS: MESA will support iMMS stakeholders by undertaking an assessment of the landscape [S24] of ongoing research and investments in iMMS. This landscaping exercise will facilitate the identification of who is doing what and where, the emerging best practices, research gaps and priority areas for funding to drive evidence-based policy development.
- Creation of a resource compilation on iMMS: The MESA Resource Hub[S22], a one-stop-shop of malaria resources, can be leveraged to facilitate dissemination of crucial iMMS resources (e.g. reference documents, training materials, online tools, protocols, videos, etc.) and democratize access to genomic data sources.
- Communities of Practices: MESA will harness its convening power to foster an effective community of practice to facilitate collaboration and sharing of knowledge.

MESA offers a dynamic and neutral platform that aims to tackle unmet challenges in malaria control and elimination, bridge knowledge gaps and drive progress to achieve the shared global vision of a malaria-free world.

***Wellcome Sanger Institute's role in supporting iMMS in Africa (Shavanthi Rajatileka from the Wellcome Sanger Institute):***  The Wellcome Sanger Institute has moved towards a decentralized approach for generating malaria genomics data. Through the Genomic Surveillance Unit (GSU), in-country support for iMMS implementation in malaria endemic countries is provided. In-Country support is provided for the end-to-end process for sample to data generation including standardized protocols for sample and metadata collection, standardized laboratory protocols, support with laboratory set-up and establishing laboratory workflows, procurement support where feasible, tailored training for individual laboratories, bespoke laboratory train-the-trainer programs, ongoing troubleshooting and technical support, as well as sample and data analysis.

The approach taken is partner-led and teams at the GSU work together with individual partners to develop bespoke implementation, support plans, tools and resources, and in close collaboration with laboratory teams tailor protocols to an individual laboratory’s requirements and available resources. This ensures the training provided, the support given, and any resources developed are fit-for-purpose in each setting. Working together with key strategic partners, the team at Wellcome Sanger Institute, has supported six laboratories in West Africa (3) and Southeast Asia (3) with standing up an amplicon sequencing based protocol to screen for drug resistance markers in *Plasmodium falciparum.* Through funding awarded by the National Institute for Health and Care Research (NIHR), the team are currently supporting the establishment of regional genomic surveillance hubs in West Africa (in Ghana and The Gambia). The Institute is committed to continuing this level of support and collaboration to ensure successful establishment and implementation of iMMS in malaria endemic regions. *Highlights of points raised by the audience*

# Summary of points raised by audience

The presentations and panel discussions generated considerable audience engagement. The points that were consistently raised and discussed included that there is a need for regional networks to facilitate prompt iMMS data sharing and capacity development, with a strong interest in maintaining and leveraging the former WHO regional network. Standardization of methods and analytical tools is also required to ensure comparability of data from different research groups, institutions and/or parts of the continent. Additionally, there is a need for an assessment of various molecular and bioinformatics tools, along with guidance on their optimal use through demonstration of use cases. Lastly, the audience stressed that increasing domestic funding to sustain iMMS is crucial, as is building capacity in Africa for iMMS, possibly through the establishment of regional hubs for sequencing.

# Take home message and future outlook for iMMS in Africa

The symposium highlighted ongoing malaria research programs on the African continent that are employing advanced molecular tools, as well as efforts and commitment from various partners and experts to expand malaria genomics infrastructure and human capital on the continent. Recurring themes from the presentations and discussions included the need to (i) expand infrastructure and technical-know-how for storing, handling, and analyzing the significant amount of malaria genomic data that are currently being generated on the continent (ii) streamline genomic research workflows–we are indeed asking similar questions about the malaria parasites and vectors albeit in different locations (iii) make the data generated from these efforts easily accessible beyond individual research programs, in particular easily accessible by and integrated into the toolbox of NMCPs (iv) sustain these efforts through securing continued funding, maintaining efforts and momentum generated through the different training programs, and lastly (v) integrating efforts throughout the continent by leveraging regional hubs, which would in turn ensure that resources are maximized.

Looking ahead, for iMMS to be successful on the African continent, current independent efforts focused on different biological components of the malaria transmission cycle must be willing to, at minimum, share resource and exchange knowledge, if not fully integrate their efforts. It is encouraging to see that we all agree on the existing malaria genomics research gaps; that we need to integrate efforts and especially work with NMCPs; and that these efforts must be sustainable. Beyond just agreeing, some practical ways that we foresee establishing sustainable iMMS on the continent include working together to develop, at a minimum: (i) well-structured guidelines for the different stages of malaria genomics workflow including how to navigate supply chain issues. This should be accompanied by training and could be agnostic of research questions to ensure wide adaptation and use (ii) user friendly “plug-and-play” data analysis tools that require minimal technical expertise to use (iii) a way to ensure that these resources and tools do not become obsolete. This will require sustainable funding, perhaps through buy-in from local governments and institutions, as well as ensuring that the resources and associated trainings remain up to date. While progress is being made, a significant amount of work remains to ensure full coverage of malaria genomics research efforts and its benefits across the African continent.

**Author contributions**

Symposium organizers and session chairs: DSI, SR, CA, & JR; Manuscript conceptualization and preparation: ND, VS, & DSI; All authors provided an abstract or summary of their contributions to the symposium, read and reviewed the original draft, and approved the final version for submission. The findings and views expressed in this article are those of the authors and do not necessarily reflect the views of their institutions or any institution referenced in the article.

# Supplementary references

S1. Etang, J. *et al.* (2022) Patterns of Kdr-L995F Allele Emergence Alongside Detoxifying Enzymes Associated with Deltamethrin Resistance in Anopheles gambiae s.l. from North Cameroon. *Pathogens* 11, 253

S2. Amplicon toolkit[Online]. Available: https://www.malariagen.net/resources/amplicon-sequencing-toolkit/. [Accessed: 15-May-2024]

S3. Ndwiga, L. *et al.* (2021) A review of the frequencies of *Plasmodium falciparum* Kelch 13 artemisinin resistance mutations in Africa. *International Journal for Parasitology: Drugs and Drug Resistance* 16, 155–161

S4. Gadalla, N.B. *et al.* (2010) Dynamics of pfcrt alleles CVMNK and CVIET in chloroquine-treated Sudanese patients infected with Plasmodium falciparum. *Malaria Journal* 9, 74

S5. Mombo-Ngoma, G. *et al.* (2011) High prevalence of dhfr triple mutant and correlation with high rates of sulphadoxine-pyrimethamine treatment failures in vivo in Gabonese children. *Malar J* 10, 123

S6. Aubouy, A. *et al.* (2003) DHFR and DHPS genotypes of Plasmodium falciparum isolates from Gabon correlate with in vitro activity of pyrimethamine and cycloguanil, but not with sulfadoxine–pyrimethamine treatment efficacy. *Journal of Antimicrobial Chemotherapy* 52, 43–49

S7. Makoni, M. (2020) Africa’s $100-million Pathogen Genomics Initiative. *The Lancet Microbe* 1, e318

S8. Inzaule, S.C. *et al.* (2021) Genomic-informed pathogen surveillance in Africa: opportunities and challenges. *The Lancet Infectious Diseases* 21, e281–e289

S9. Moyo, E. *et al.* (2023) Implementation of Public Health Genomics in Africa: Lessons from the COVID-19 pandemic, challenges, and recommendations. *Journal of Medical Virology* 95, e28295

S10. Ramsay, M. (2022) African genomic data sharing and the struggle for equitable benefit. *Patterns (N Y)* 3, 100412

S11. Wonkam, A. *et al.* (2022) Five Priorities of African Genomics Research: The Next Frontier. *Annual Review of Genomics and Human Genetics* 23, 499–521

S12. Mensah, B.A. *et al.* (2022) Genomic approaches for monitoring transmission dynamics of malaria: A case for malaria molecular surveillance in Sub–Saharan Africa. *Frontiers in Epidemiology* 2

S13. Sy, M. *et al.* (2022) Plasmodium falciparum genomic surveillance reveals spatial and temporal trends, association of genetic and physical distance, and household clustering. *Sci Rep* 12, 938

S14. Mayor, A. *et al.* (2023) Sampling for malaria molecular surveillance. *Trends in Parasitology* 39, 954–968

S15. Home | Pan-African Mosquito Control Association[Online]. Available: https://www.pamca.org/en. [Accessed: 15-May-2024]

S16. MalariaGEN vector data user guide — MalariaGEN vector data user guide[Online]. Available: https://malariagen.github.io/vector-data/landing-page.html. [Accessed: 29-Jul-2024]

S17. Use cases for NMCP’s[Online]. Available: https://www.malariagen.net/resources/use-cases-for-nmcps/. [Accessed: 15-May-2024]

S18. Tools[Online]. Available: https://genremekong.org/tools. [Accessed: 29-Jul-2024]

S19. grcMalaria R package - User Guide. *grcMalaria R package - User Guide*. [Online]. Available: https://genremekong.org/tools/grcmalaria-r-package-user-guide. [Accessed: 29-Jul-2024]

S20. Pf7: An open dataset of Plasmodium falciparum genome variation in 20,000 worldwide samples[Online]. Available: https://www.malariagen.net/resource/34/. [Accessed: 29-Jul-2024]

S21. Bill & Melinda Gates Foundation | Bill & Melinda Gates Foundation[Online]. Available: https://www.gatesfoundation.org/. [Accessed: 29-Jul-2024]

S22. adttsmes Home. *MESA*. [Online]. Available: https://mesamalaria.org/. [Accessed: 15-May-2024]

S23. Alonso, P.L. *et al.* (2011) A Research Agenda to Underpin Malaria Eradication. *PLOS Medicine* 8, e1000406

S24. Malaria Molecular Surveillance (MMS). *MESA*. [Online]. Available: https://mesamalaria.org/mesa-track/deep-dives/malaria-molecular-surveillance-mms/
